# Supplementary material for: A roadmap for the computation of persistent homology
Source: EPJ Data Sci. 2017 Aug 9;6(1):17. doi: 10.1140/epjds/s13688-017-0109-5 (PMC6979512; doi:10.1140/epjds/s13688-017-0109-5)
Supplement: Supplementary file 1 — Additional computations. (pdf) [file 13688_2017_109_MOESM1_ESM.pdf]

ADDITIONAL COMPUTATIONS

1. Introduction. We report additional computations that we perform with the libraries that we study. See the main paper for a description of the data sets and details on how we perform the computations.

2. Vietoris–Rips complex. In this section, we report additional results for the computation of PH with the VR complex, as explained in Section 7 of the main manuscript. In Tables 1–4, we give results for the computations on a cluster; in Table 5, we give results for the computations on a shared-memory system. For the data set **Vicsek**, we computed PH for six different point clouds, as we also explain in the main paper. We report results only for one of these points clouds, because the results of the computations — in terms of wall-time seconds and memory used — were all similar. The point cloud for which we report the results has the following parameters (see the main manuscript for a discussion of these parameters): $N = 300$, $l = 5$, $\theta_0 = 1$, $v_0 = 0.03$, $\eta = 0.1$, and $T = 600$. Of the 600 different points clouds (one for each time step t), we choose the one that corresponds to time step $t = 300$.

Table 1

*Computations of PH with VR complex for the data set **drag 1**. The size of the complex is* 1.7×10^8*, and the dimension is* 2*. We run* DIPHA *on one node and* 16 *cores.*

	Wall-time seconds	CPU seconds	Memory in GB
javaPlex (st)	-	-	> 64
Dionysus (st)	-	-	-
DIPHA (st)	7356	117417	2.4
Perseus	-	-	-
Dionysus (d)	4360	4362	16.8
DIPHA (d)	75	1176	1.8
GUDHI	1524	1517	7.9
Ripser	2	1	0.04

Table 2

*Computations of PH with VR complex for the data set **house**. The size of the complex is* 1.6×10^9*, and the dimension is* 3*. We run* DIPHA *(d) on three nodes of* 16 *cores, and* DIPHA *(st) on four nodes of* 16 *cores.*

	Wall-time seconds	CPU seconds	Memory in GB
javaPlex (st)	-	-	> 64
DIPHA (st)	53686	243587	5.3
Perseus	-	-	-
Dionysus (d)	-	-	-
DIPHA (d)	1450	22981	7.1
GUDHI	46377	25925	64.6
Ripser	11	11	0.8

3. Image data set. In this section, we report timings and memory usage for the computation of PH with cubical complexes with the libraries DIPHA, Perseus, and GUDHI for the data set **vertebra**. For this data set, the standard implementation in DIPHA performs better in terms of wall time, CPU seconds, and memory usage than the dual implementation. This does not necessarily contradict the fact that the dual implementation gives a heuristic speed-up with respect to the standard imple-

Table 3

*Computations of PH with VR complex for the data set **senate**. The size of the complex is 4.6×10^6, and the dimension is 3. We run DIPHA on two nodes and 16 cores.*

	Wall-time seconds	CPU seconds	Memory in GB
javaPlex (st)	222	571	< 5
DIPHA (st)	5	58	0.1
Perseus	457	457	4.9
Dionysus (d)	183	183	0.5
DIPHA (d)	5	34	0.1
GUDHI	51	51	0.2
Ripser	1	1	0.01

Table 4

*Computations of PH with VR complex for the data set **netw-sc**. The size of the complex is 8.5×10^8, and the dimension is 3. We run DIPHA on two nodes of 16 cores.*

	Wall-time seconds	CPU seconds	Memory in GB
javaPlex (st)	-	-	> 64
DIPHA (st)	16004	202755	5.6
Perseus	-	-	-
Dionysus (d)	-	-	-
DIPHA (d)	618	9842	5.8
GUDHI	13483	13465	40.6
Ripser	9	9	0.5

Table 5

Computations on the shared-memory system.

Data set	**H3N2**	**Vicsek**	**drag 1**	**fract l**
Size of complex	3.4×10^9	3.3×10^8	1.7×10^8	2.8×10^9
Max. dim.	2	3	2	3
javaPlex (st)	-	26257	19054	-
Perseus	-	-	-	-
Dionysus (d)	-	4637	2889	593528
DIPHA (d)	14362	369	1775	3920
GUDHI	3377	433	144	4154
Ripser	24	3	2	14

(a) Computations on the shared-memory system: wall-time seconds

Data set	**H3N2**	**Vicsek**	**drag 1**	**fract l**
Size of complex	3.4×10^9	3.3×10^8	1.7×10^8	2.8×10^9
Max. dim.	2	3	2	3
javaPlex (st)	> 700	< 600	< 600	> 700
Perseus	-	-	-	-
Dionysus (d)	-	33.5	16.8	269
DIPHA (d)	276.1	32.5	13.8	276.1
GUDHI	158.2	15.9	7.9	134.5
Ripser	0.2	0.3	0.04	1.2

(b) Computations on the shared-memory system: memory in GB

mentation, because it is well-known that the dual implementation gives a speed-up when one computes PH with the VR complex but not in general.

TABLE 6

*Computations of PH with cubical complexes for the data set **vertebra** on the shared-memory system. The complex, as constructed by* DIPHA *and* GUDHI*, has* 1.1×10^9 *cells, and its dimension is* 3*. Note that* PERSEUS *implements a less efficient way for building filtered cubical complexes from grey-scale images, and the resulting complex has more cells than the one constructed by* DIPHA *and* GUDHI*.*

	DIPHA (d)	DIPHA (st)	PERSEUS	GUDHI (d)
Wall-time seconds	2115	1793	19604	3668
Memory in GB	82.1	80.9	628.8	59.8

4. Additional functionalities. In this section, we report timings and memory usage for the computation of PH with the alpha (α), Čech (Č), weak witness complex (W), and parametrized witness complex for $\nu = 2$ (W_2) (see the main text for a description of these complexes). For the witness complexes we choose the landmark points uniformly at random. We use the two data sets consisting of point clouds in $\mathbb{R}^3$ (i.e., the data sets **Klein** and **Vicsek**). Additionally, note that the newest version of GUDHI (Version 1.3.1) implements the computation of witness complexes, but it does not implement the computation of filtered witness complexes.

TABLE 7

Computations of alpha and Čech complexes on the shared-memory system.

	DIONYSUS α	DIONYSUS Č	GUDHI α
Size of complex	9.2×10^3	-	9.2×10^3
Wall-time seconds	1	-	2
Memory in GB	0.008	-	0.006

(a) Time and memory usage for the data set **Klein**.

	DIONYSUS α	DIONYSUS Č	GUDHI α
Size of complex	7.7×10^3	-	7.7×10^3
Wall-time seconds	1	-	2
Memory in GB	0.007	-	0.006

(b) Time and memory usage for the data set **Vicsek**. For the witness complexes we choose 100 landmark points uniformly at random.

Table 8

Computations of PH with the witness complex on the shared-memory system.

	javaPlex W	javaPlex W_2
Size of complex	6.8×10^5	2.8×10^6
Wall-time seconds	32	328
Memory in GB	< 2	< 5

(a) Time and memory usage for the data set **Klein**. We choose 100 landmark points uniformly at random.

	javaPlex W	javaPlex W_2
Size of complex	1×10^6	4.1×10^6
Wall-time seconds	50	84
Memory in GB	< 3	< 5

(b) Time and memory usage for the data set **Vicsek**. For the witness complexes, we choose 100 landmark points uniformly at random.
