# Supplementary material for: A roadmap for the computation of persistent homology
Source: EPJ Data Sci. 2017 Aug 9;6(1):17. doi: 10.1140/epjds/s13688-017-0109-5 (PMC6979512; doi:10.1140/epjds/s13688-017-0109-5)
Supplement: Supplementary file 2 — Tutorial for ‘A roadmap for the computation of persistent homology’. (pdf) [file 13688_2017_109_MOESM2_ESM.pdf]

Tutorial for "A roadmap for the computation of persistent homology"

1 Introduction

In this tutorial, we give detailed guidelines for the computation of persistent homology and for several of the functionalities that are implemented by the libraries in Table 2 in the main manuscript. We first give some advice on how to install the various libraries, and we then give guidelines for how to compute PH for every step of the pipeline in Fig. 3 of the main paper. We explain how to compute PH for networks with the weight rank clique filtration (WRCF); for point clouds with the VR, alpha, Čech, and witness complexes; and for image data sets with cubical complexes. We then give guidelines for visualizing the outputs of the computations and for computing the bottleneck and Wasserstein distances with DIONYSUS and HERA. In addition to the bottleneck and Wasserstein distances, there are also other tools (such as persistence landscapes and confidence sets) that are useful for statistical assessment of barcodes, but we do not discuss them here, as there are already comprehensive tutorials [4,5] for the packages that implement these methods. All MATLAB scripts written for this tutorial are available at `https://github.com/n-otter/PH-roadmap/tree/master/matlab`. In Fig. 1, we give instructions for how to navigate this tutorial.

Many tears and much sweat and blood were spent learning about the different libraries, writing this tutorial, and the scripts. If you find this tutorial helpful, please acknowledge it.

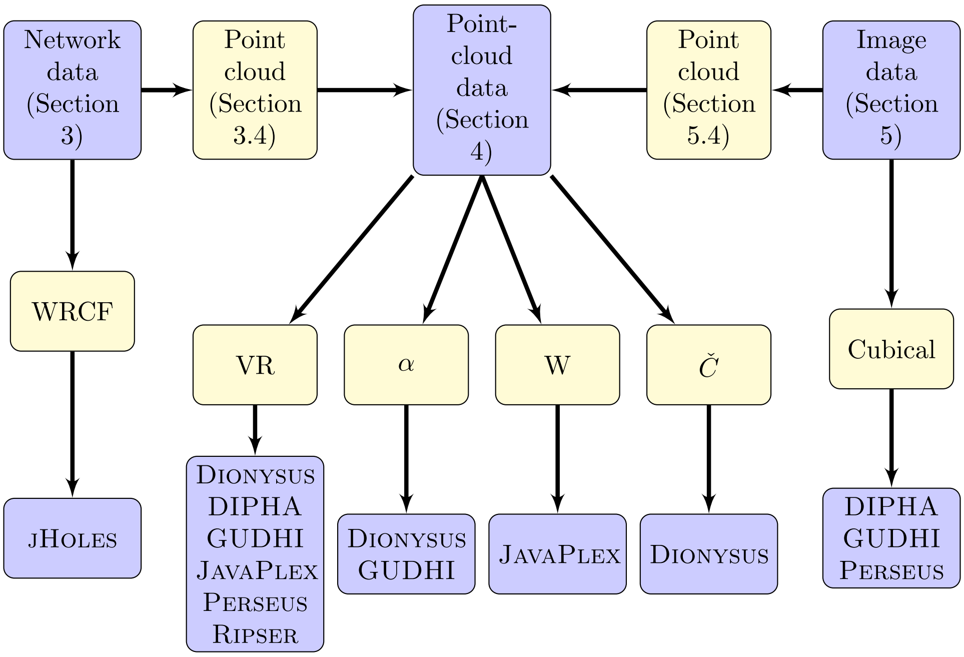

Figure 1: Schematic for how to navigate the tutorial.

Contents

1 Introduction **1**

2 Installation **3**
2.1 Dionysus . . . 3
2.2 DIPHA . . . 3
2.3 GUDHI . . . 3
2.4 JavaPlex . . . 3
2.5 Hera . . . 4
2.6 jHoles . . . 4
2.7 Perseus . . . 4
2.8 Ripser . . . 4

3 Computation of PH for networks **4**
3.1 Sample network data . . . 4
3.2 Adjacency matrix versus edge-list file . . . 5
3.3 PH with the WRCF (with jHoles) . . . 5
3.4 Networks as point clouds . . . 5

4 Computation of PH for point clouds **6**
4.1 Sample point-cloud data . . . 6
4.2 Distance matrices versus points clouds . . . 6
4.3 VR complex . . . 6
4.3.1 Input data . . . 6
4.3.2 Dionysus . . . 7
4.3.3 DIPHA . . . 8
4.3.4 GUDHI . . . 8
4.3.5 JavaPlex . . . 8
4.3.6 Perseus . . . 9
4.3.7 Ripser . . . 9
4.4 Alpha . . . 10
4.4.1 Dionysus . . . 10
4.4.2 GUDHI . . . 10
4.5 Čech . . . 11
4.6 Witness . . . 11

5 Computation of PH for image data **11**
5.1 DIPHA . . . 12
5.2 Perseus . . . 12
5.3 GUDHI . . . 12
5.4 Images as point clouds . . . 13

6 Barcodes and persistence diagrams **13**

7 Statistical interpretation of barcodes **14**
7.1 Bottleneck distance . . . 15
7.1.1 Dionysus . . . 15
7.1.2 Hera . . . 15
7.2 Wasserstein distance . . . 16
7.2.1 Dionysus . . . 16
7.2.2 Hera . . . 16

2 Installation

In this section, we give guidelines on how to get and/or install the software packages.

2.1 Dionysus

The code for DIONYSUS is available at `http://www.mrzv.org/software/dionysus/get-build-install.html`, where one can also find information on dependencies and how to build the library.

The library is written in C++, but it also supports python bindings (i.e., there is a python interface to some of the functionalities that are implemented in the library). Depending on the machine on which one is building DIONYSUS, the python bindings can create some issues. Additionally, from the perspective of performance, it is better to directly use the C++ code. If one wishes to build the library without the python bindings, one needs to delete the `bindings` directory and also to delete the (last) line `add_subdirectory (bindings)` in the file `CMakeLists.txt`.

If one seeks to compute the bottleneck or Wasserstein distances with the library (see Section 7), then before building the library, one needs to amend a mistake in the bottleneck-distance.cpp script as follows: in the subdirectory `examples`, one finds the file `bottleneck-distance.cpp`. One needs to uncomment the following line (which occurs towards the end of the file):

```
std::cout << "Distance: " << bottleneck_distance(dgm1, dgm2) << std::endl;
```

Now one can build the library as follows (from the directory in which the `CMakeLists.txt` file is):

```
$ mkdir build
$ cd build
$ cmake ..
$ make
```

2.2 DIPHA

The DIPHA library is available at `https://github.com/DIPHA/dipha`. One can build the library as follows (from the directory in which the `CMakeLists.txt` file is):

```
$ mkdir build
$ cd build
$ cmake ..
$ make
```

2.3 GUDHI

The GUDHI library is available at `https://gforge.inria.fr/frs/?group_id=3865`. Information about dependencies and how to build the library is available at `http://gudhi.gforge.inria.fr/doc/latest/installation.html`. One can build the library in a similar way as explained for DIPHA.

We note that a python interface was released with the most recent version (at the time of this writing) of the library GUDHI; in this tutorial, we give instructions on how to use the C++ implementation, and we point readers who are familiar with python to the documentation available at `http://gudhi.gforge.inria.fr/python/latest/`.

2.4 JavaPlex

The JAVAPLEX library does not require installation or to be built, and all implementations that we listed in Table 2 in the main text can be found in the directory `matlab-examples_x.y.z` (where `x.y.z` stands for the version number). This directory, and the accompanying tutorial, can be downloaded at `https://github.com/appliedtopology/javaplex/releases/`. All of the scripts in `matlab-examples_x.y.z` are written in MATLAB.

2.5 Hera

The HERA library is available at `https://bitbucket.org/grey_narn/hera`. The root folder includes two subfolders: `geom_bottleneck` contains the source code for computing bottleneck distance, and `geom_matching` contains the source code for computing Wasserstein distance.

One can build the library by running the following commands in each of the subfolders `geom_bottleneck/` and `geom_matching/wasserstein`:

```
$ mkdir build
$ cd build
$ cmake ..
$ make
```

2.6 jHoles

Ideally, the JHOLES library should be available for download at `http://cuda.unicam.it/jHoles`. However, this website is often down, so the best way to obtain the library is to contact Matteo Rucco, who is the corresponding author of the companion paper [3]. The library does not require installation or to be built.

2.7 Perseus

A compiled version of the PERSEUS library is available at `http://people.maths.ox.ac.uk/nanda/perseus/`. Those wishing to build the library from source code can find the source code at the same website.

2.8 Ripser

The RIPSER library is available at `https://github.com/Ripser/ripser`. One can build the library by running `make` in the folder that includes the `Makefile`. Note that RIPSER supports several options that can be passed to `make`. See `https://github.com/Ripser/ripser` for more information.

3 Computation of PH for networks

In this section, we explain how to compute PH for undirected weighted networks. We represent the nodes of a network with N nodes using the natural numbers $1, \ldots, N$.

3.1 Sample network data

To create weighted networks, one can use the script `fractal_weighted.m` available at `https://github.com/n-otter/PH-roadmap/tree/master/matlab/synthetic_data_sets_scripts`. We recall that a fractal network is determined by three non-negative integers n, b and k (see the main paper for details). The script `fractal_weighted.m` takes four parameters as input: (1) a natural number n (the total number of nodes of the graph is 2^n); (2) a natural number b (the number of nodes of the initial network); (3) a natural number k (this is the connection density parameter); and (4) a string which indicates how weights are associated to edges: this is either `'random'`or `'linear'` (see the main paper for details).

Example: The command

```
>> fractal_weighted(4,2,1,'random')
```

saves the files `fractal_4_2_1_random.txt` and `fractal_4_2_1_random_edge_list.txt`, where the first file is a text file storing the weighted adjacency matrix of a fractal network with 16 nodes and random weight on every edge, while the second file is a text file storing the weighted edge list of the same network.

3.2 Adjacency matrix versus edge-list file

We assume that a network is given either as an adjacency matrix in a MAT-file or as a text file with a list of weighted edges. A typical entry on one line of such a file is a triple "$i\ j\ w_{ij}$", where i and j are the nodes incident to the edge and w_{ij} is the weight of the edge. We call such a file an "edge-list file". We provide the script adj_matrix_to_edge_list.m to obtain edge-list files from adjacency matrices. (Note that we also provide the script edgelist_to_point_cloud_dist_mat.m to obtain distance matrices from edge-list files, where the distances between nodes are computed using shortest paths.)

3.3 PH with the WRCF (with jHoles)

Using edge-list files, we compute PH by constructing the weight rank clique filtration (WRCF) with the library jHoles. Here we give instructions on how to compute PH with Version 3 of jHoles. One needs to run the following command in the terminal:

```
$ java -Xmx<value> -jar jHoles.jar input-file output-file1 output-file2
```

where -Xmx<value> is optional and can be used to set the maximum heap size of the garbage collector (we recommend doing this for networks with a large number of nodes or high density). For example, -Xmx4g sets the maximum heap size to 4 gigabytes. The file input-file is the edge-list file of the network, and output-file1 is a file in which information (e.g., number of edges, average degree, etc.) about the network is saved, and output-file2 is the file in which the intervals are saved.

Example: With the command

```
$ java -Xmx4g -jar jHoles.jar fractal_4_2_1_random_edge_list.txt fractal_info.txt \
 fractal_intervals.txt
```

one computes PH with the WRCF for the fractal network with parameters $(n, b, k) = (4, 2, 1)$ and random weight on every edge. The persistence diagram is saved in the file fractal_intervals.txt, where for every interval also representative cycles are given, while in file fractal_info.txt information such as average degree, density, or average cluster is given. Note that the backslash in the above command indicates that the command continues on the next line, and should therefore be omitted if one writes the whole command on the same line.

3.4 Networks as point clouds

One can construe a connected weighted network as a finite metric space and then compute PH by using one of the methods from Section 4. We now explain how to compute a distance matrix from an undirected weighted network using information about shortest paths between nodes. If two nodes i and j are connected by an edge with weight w, we set the distance between i and j to be $1/w$. Otherwise, we define the distance between i and j to be the minimum of the lengths of all paths between them, where we define the length of a path to be the the sum of the inverse of the weights of the edges in this shortest path. One can compute this distance matrix with the script shortest_paths.m. As input, it takes an edge-list file. (See Section 3.2 for how to obtain an edge-list file from a MAT-file that stores an adjacency matrix.) The output of the script is a text file in which each line gives the entries of a row of the distance matrix. (Note that if a network is not connected, then one sets the distance between nodes in two distinct components of the network to be infinite, and one thereby obtains an extended metric space.)

Example:

```
>> shortest_paths('fractal_4_2_1_random_edge_list.txt')
```

saves the text file 'fractal_4_2_1_random_edge_list_SP_distmat.txt'.

Additionally, using tools like multidimensional scaling, one can convert a distance matrix into a finite set of points in Euclidean space. This is handy if one wants to use a library that does not support distance matrices as an input type. We provide the script `distmat_to_pointcloud.m` to obtain a point cloud from a distance matrix using multidimensional scaling.

Example:

```
>> distmat_to_pointcloud('fractal_4_2_1_random_edge_list_SP_distmat.txt')
```

4 Computation of PH for point clouds

In this section, we explain how to compute PH for finite metric spaces.

4.1 Sample point-cloud data

We provide scripts to create point-cloud data. These are available at `https://github.com/n-otter/PH-roadmap/tree/master/matlab/synthetic_data_sets_scripts`. To create points clouds in $\mathbb{R}^3$ and $\mathbb{R}^4$, one can use the scripts `klein_bottle_imm.m` and `klein_bottle_emb.m`, respectively.

Example:

```
>> klein_bottle_imm(5)
```

samples 25 points uniformly at random from the image of the immersion of the Klein bottle in $\mathbb{R}^3$ and saves the point cloud in the text file `klein_bottle_pointcloud_25.txt`, with each line storing the coordinates of one point, as well as in the MAT-file `klein_bottle_25.mat`.

4.2 Distance matrices versus points clouds

Given a finite set of points in Euclidean space, one can compute an associated distance matrix. To get a distance matrix from a point cloud, we provide the script `pointcloud_to_distmat.m`.

Example:

```
pointcloud_to_distmat('klein_bottle_pointcloud_25.txt')
```

computes the distance matrix for the 25 points sampled from the Klein bottle and saves it in the text file `'klein_bottle_pointcloud_25_distmat.txt'`.

Conversely, a distance matrix can yield a finite set of points in Euclidean space by using a method such as multidimensional scaling. We implement such a conversion in the script `distmat_to_pointcloud.m`.

Example:

```
>> distmat_to_pointcloud('klein_bottle_pointcloud_25_distmat.txt')
```

4.3 VR complex

4.3.1 Input data

The standard input for the construction of the VR complex is a distance matrix. The software packages that take a distance matrix as input are Perseus and DIPHA, but the other packages do not. Instead, they take a set of points in Euclidean space as input. Note that Perseus can also take a set of points in Euclidean space as input, but the implementation does not allow one to set a bound on the upper dimension in the computation of the VR complex, so this implementation is impractical to use for most data sets. We next compute the VR complex with each library. With most of the libraries, one has to indicate the

maximum filtration value for which one wants to compute the filtered simplicial complex. We set this value to the maximum distance between any two points. Note, however, that a smaller value often suffices. To compute the maximum distance given a text file `input-file` with the coordinates of a point on each line, one can type the following in MATLAB:

```
>> A=load(input-file);
>> D=pdist(A);
>> D=squareform(D);
>> M=max(max(D));
```

Example:

```
>> A = load('klein_bottle_pointcloud_25.txt');
>> D=pdist(A);
>> D=squareform(D);
>> M=max(max(D));
>> M

M =

    7.0513
```

The maximum distance is given by `M`. Similarly, if one is given a text file `input-file` that stores a distance matrix, then one can compute the maximum distance by typing

```
>> D=load(input-file);
>> M=max(max(D));
```

4.3.2 Dionysus

The library DIONYSUS takes a point cloud as input. We can use either the standard or dual algorithm. For the former, we use the command

```
$ ./rips-pairwise -s max-dimension -m max-distance \
-d output-file input-file
```

where the file `./rips-pairwise` is in the `dionysus/build/examples/rips` directory, further `max-dimension` is the maximum dimension for which we want to compute the simplicial complex, `max-distance` is the maximum parameter value for which we want to compute the complex, `output-file` is the file to which the intervals are written, and `input-file` is a text file with the coordinates of a point on each line. To compute the intervals with the dual algorithm, we use the command

```
$ ./rips-pairwise-cohomology -s max-dimension -m max-distance \
-p prime -d output-file input-file
```

where the file `./rips-pairwise-cohomology` is in the `dionysus/build/examples/cohomology` directory, further `prime` is a prime number and indicates the coefficient field for the computation of cohomology. (For the standard algorithm, there is no choice: one must use $p = 2$.)

Example:

```
$  ./rips-pairwise-cohomology -s 3 -m 7.0513 -p 2 -d klein_bottle_25_output.txt \
klein_bottle_pointcloud_25.txt
```

4.3.3 DIPHA

The DIPHA library reads and writes to binary files. One can convert the text file that stores the distance matrix (see Section 4.2 for how to obtain a distance matrix from a point cloud) into a binary file of the right input type for DIPHA by using the file `save_distance_matrix.m` provided by the developers of DIPHA, which can be found in `dipha-master/matlab`.

Example:

```
>> D=load('klein_bottle_pointcloud_25_distmat.txt');
>> save_distance_matrix(D,'kleinbottle_25.bin');
```

To run DIPHA using a single process, one types the command

```
$ ./dipha [options] --upper_dim d input-file output-file
```

where options include `--benchmark` to display profiling information and `--dual` to run the dual algorithm. (The default is the standard algorithm.) To run DIPHA on more than one process, one uses the command

```
$ mpiexec -n N dipha options --upper-dim d input-file output_file
```

where `N` is the number of process and the above options are again available.

Example:

```
$ ./dipha --benchmark --dual --upper_dim 3 klein_bottle_25.bin klein_bottle_out.bin
```

4.3.4 GUDHI

The library GUDHI takes a point cloud as input. We run the command

```
$ ./rips_persistence -r max-distance -d max-dimension \
-p prime -o output-file input-file
```

where `max-distance`, `max-dimension`, and `prime` are as above, `output-file` is a file to which the intervals are written, and `input-file` is a text file with the coordinates of a point on each line.

Example:

```
$ ./rips_persistence -r 7.0513 -d 3 -p 2 -o klein_bottle_25_output.txt \
 klein_bottle_pointcloud_25.txt
```

4.3.5 JavaPlex

The library JavaPlex takes a point cloud as input. Before starting using this library one has to run the script `load_javaplex.m` which is located in the JavaPlex directory `matlab_examples`. We wrote the script `vietoris_rips_javaplex.m` to compute PH with the VR complex in JavaPlex. This script takes four parameters as input: (1) the name of the text file with the point cloud; (2) the maximum dimension for which we want to compute the simplicial complex; (3) the maximum filtration step for which we want to compute the VR complex; and (4) the number of filtration steps for which we compute the VR complex. The script saves text files containing the barcode intervals, one file for each homological dimension. These are the files ending with `i_right_format.txt` where i indicates the homological dimension.

Example:

```
>> vietoris_rips_javaplex('klein_bottle_pointcloud_25.txt',7.0513,3,20);
```

4.3.6 Perseus

The library PERSEUS takes a distance matrix as input (see Section 4.2 for how to obtain a distance matrix from a point cloud). One has to prepare the input file by adding two lines at the beginning of the file that stores the distance matrix. We do this as follows:

```
N
first-step step-increment steps max-dimension
d11 d12 ...
d21 d22 ...
...
```

where `N` is the number of points (and hence the number of rows (or columns) in the distance matrix), `first-step` is the value for the first filtration step, `step-increment` is the step size between any two filtration steps, `steps` is the number of total steps, and `max-dimension` is the maximum dimension for which we compute the complex. We now can compute PH with the command

```
$ ./perseus distmat input-file output-file
```

in the terminal, where `input-file` is the name of the input file and `output-file` is the name of the file in which the intervals will be saved. PERSEUS creates a series of files named `output-file_i.txt` for $i \in \{0, 1, \dots\}$, where `output-file_i.txt` contains the intervals for the homological degree i.

Example:

```
$ ./perseus distmat klein_bottle_25.txt klein_bottle_25_output.txt
```

where these are the first two lines of the file `klein_bottle_25.txt`:

```
25
0 0.1 71 3
```

4.3.7 Ripser

The library RIPSER takes both a point cloud and a distance matrix as input, and it supports four different format types for the distance matrix. (See `https://github.com/Ripser/ripser#description` for more details.) One of the supported input types for the distance matrix is the format accepted by DIPHA (see Section 4.3.3).

We run the command

```
$ ./ripser --format input-type --dim max-dimension [options] input-file
```

where `input-type` is a string that indicates the type of the input, `max-dimension` is the maximum dimension of persistent homology that is computed (note the difference with respect to the other libraries, for which one indicates the maximum dimension of the complex), and options includes `-- modulus p` (with which one can choose the coefficient field $\mathbb{F}_p$). Note that one has to enable this option at compilation (see Section 2.8). The output of the computation is written to the standard output.

Example:

```
$ ./ripser --format dipha --dim 2 klein_bottle_25.bin > klein_bottle_25_out.log
```

where `klein_bott_25.bin` is the input file from Section 4.3.3 and the standard output is saved to the file `klein_bottle_25_out.log`.

4.4 Alpha

In this section, we explain how to compute PH with the alpha complex with Dionysus and GUDHI.

4.4.1 Dionysus

One can compute PH with the alpha complex for finite subsets of points in $\mathbb{R}^2$ or $\mathbb{R}^3$. For points clouds in $\mathbb{R}^2$, one runs the command

```
$ ./alphashapes2d < input-file > output-file
```

where `input-file` a text file with coordinates of a point in $\mathbb{R}^2$ on each line and `output-file` is the file to which the intervals in the persistence diagram are written. For points clouds in $\mathbb{R}^3$, one runs the command

```
$ ./alphashapes3d-cohomology input-file output-file
```

where `input-file` and `output-file` are as above.[1]

Example:

```
$ ./alphashapes3d-cohomology klein_bottle_pointcloud_25.txt klein_bottle_25_output.txt
```

4.4.2 GUDHI

The program GUDHI supports both points clouds in $\mathbb{R}^2$ and $\mathbb{R}^3$. To compute PH with the alpha complex one can use the script `./alpha_complex_persistence` which is in the folder `example/Persistent_cohomology`. The script takes as input an OFF file, as decribed here `http://www.geomview.org/docs/html/OFF.html`. Namely, the first lines of the input file are as follows:

```
OFF
embedding-dimension V 0 0
x11 x12 ... x1d
x21 x22 ... x2d
...
```

where `embedding-dimension` is the dimension d of the Euclidean space, `V` is the number of points, and all other lines store coordinates `xi1`,..., `xid` of the points.

One then computes PH by running the following command in the terminal

```
$ ./alpha_complex_persistence -p prime -o output-file input-file
```

where `p` is a prime number and indicates that one does computations over the coefficient field $\mathbb{F}_p$.

Example:

```
$ ./alpha_complex_persistence  -p 2 -o klein_bottle_25_output.txt klein_bottle_25_input.txt
```

where the first two lines of the file `klein_bottle_25_input.txt` are as follows:

```
OFF
3 25 0 0
```

[1] There is also a script `./alphashapes3d` , but this script has a bug and does not compute.

4.5 Čech

One can compute PH with the Čech complex for a point cloud in Euclidean space using the implementation in Dionysus. One runs the command

```
$ ./cech-complex < input-file > output-file
```

where `input-file` is a text file of the following form:

```
embedding-dimension max-dimension
x11 x12 ... x1d
x21 x22 ... x2d
...
```

where `embedding-dimension` is the dimension d of the Euclidean space, `max-dimension` is the dimension up to which we compute the complex, and all other lines store coordinates `xi1`,..., `xid` of the points.

Example:

```
$ ./cech-complex < klein_bottle_25_input.txt > klein_bottle_output.txt
```

where the first line of the file `klein_bottle_25_input.txt` is as follows:

```
3 3
```

4.6 Witness

One can compute the witness complex using JavaPlex. Recall that before starting using this library one has to run the script `load_javaplex.m` which is located in the JavaPlex directory `matlab_examples`. Given a point cloud S, the witness complex is a simplicial complex constructed on a subset $L \subseteq S$ of so-called "landmark" points. As we explained in the main manuscript, there are several versions of the witness complex. The ones implemented in JavaPlex are the weak Delaunay complex, which is also just called the "witness complex", and parametrized witness complexes, which are also known as "lazy witness complexes". Given a point cloud L, one can compute the witness complex or lazy witness complex using the scripts `witness_javaPlex.m` and `lazy_witness_javaPlex.m`.

The script `witness_javaPlex.m` takes four parameters as input: (1) the name of the text file with the point cloud; (2) the maximum dimension for which we want to compute the simplicial complex; (3) the maximum filtration value for which we want to compute PH; and (4) the number of filtration steps for which we compute the complex.

The script `lazy_witness_javaPlex.m` takes six parameters as input: (1) the name of the text file with the point cloud; (2) the maximum dimension for which we want to compute the simplicial complex; (3) the number of landmark points; (4) how the landmark points are selected (this is either `'random'` or `'maxmin'`); (5) the value for the parameter ν; and (6) the number of filtration steps for which we compute the complex.

See the scripts for further details on input parameters, and see the main manuscript and the JavaPlex tutorial [2] for further detail on witness complexes.

Example:

```
lazy_witness_javaPlex('klein_bottle_pointcloud_25.txt',3,10,'random',2,20)
```

5 Computation of PH for image data

In this section, we discuss how to compute PH for image data using cubical complexes. The packages DIPHA, Perseus, and GUDHI support the construction of filtered cubical complexes from grey-scale image data. As an example of grey-scale image data, we use the data set "Nucleon" from the Volvis

repository [1]. This is a 3-dimensional grey-scale image data set; one is given a 3-dimensional lattice of resolution $41 \times 41 \times 41$, where each lattice point is labeled by an integer that represents the grey-scale value for the voxel anchored at that lattice point. The .raw data file from [1] is binary, and it stores 8 bits for each voxel. We read the .raw data file in MATLAB as follows:

```
>> fileID=fopen('nucleon.raw','r');
>> A=fread(fileID,41*41*41,'int8');
>> B=reshape(A,[41 41 41]);
```

so `B` is a 3-dimensional array of size $41 \times 41 \times 41$ that stores the grey-scale values.

Note for this example that the cubical complex constructed in DIPHA and GUDHI has dimension 3 and size 531441, while the cubical complex constructed with Perseus has dimension 3 and size 571787. This is because DIPHA and GUDHI implement the optimized way to represent a cubical complex that was introduced in [7]. However, all three libraries implement the same algorithm for the computation of PH from cubical complexes. When interpreting the results of the computations with GUDHI and Perseus, one needs to take into account the rescaling of the grey values (see Section 5.2).

5.1 DIPHA

To save the array in a file that can be given as input to DIPHA, one can use the MATLAB script `save_image_data.m` provided by the developers of DIPHA, which can be found in `dipha-master/matlab`. One gives the array `B` as input and a name for the input file. One then proceeds in a similar way as for the computation of the VR complex (see Section 4.3).

Example:

```
>> save_image_data(B,'nucleon.bin');
$ ./dipha --benchmark nucleon.bin nucleon_out.bin
```

5.2 Perseus

To compute PH with cubical complexes with Perseus, one needs to rescale the grey values so that all grey values are positive, because Perseus does not allow cells to have negative birth times. We wrote the script `save_image_data_perseus.m` to save the array in a file that can be given as input to Perseus. This script takes the array `B` as input and a name for the input file for Perseus.

Example:

```
>> save_image_data_perseus(B,'nucleon.txt');
```

To compute PH with Perseus one then runs the following command:

```
$ ./perseus cubtop input-file output-file
```

where `input-file` is the text file prepared with the script `save_image_data_perseus.m`, and `output-file` is the name of the text file to which the barcode intervals are written.

Example:

```
$ ./perseus cubtop nucleon.txt nucleon_output.txt
```

5.3 GUDHI

To compute PH with GUDHI, one can use the same input file as for Perseus. We run the command

```
$ ./Bitmap_cubical_complex input-file
```

and the output is then saved to a file with name `input-file_persistence`.

5.4 Images as point clouds

As we discussed in Section 5.1 of the main manuscript, one can construe a collection of images as a metric space, and one can then apply the methods for computing the PH for point clouds that we discussed in Section 4.

6 Barcodes and persistence diagrams

Once we have computed the intervals, we plot the barcodes and persistence diagrams. The format of the output files varies widely across the different packages. To address this issue and to interpret the results of the computations, we first need to change the format of the output files to a common format. In the unified format, each homological dimension has an accompanying text file in which we store the intervals. In this file, the entry in line i has the form

$$x_i \; y_i \,,$$

where x_i is the left endpoint of the interval and y_i is the right endpoint. If the interval is infinite, we set $y_i = -1$.

- Dionysus: We provide the script `dionysus_reformat_output.m` to obtain the right format. This script takes two parameters as input, namely the name of the text file to which the ouput of the computations with Dionysus were stored, and a string of five letters indicating the type of file: "dcech" for the output of PH computation with the Čech complex; "alpha" for the output of PH computation with the alpha complex; "VR-st" for the output of PH computation with a Vietoris–Rips complex and the standard algorithm; and "VR-co' for the output of PH computation with a Vietoris–Rips complex and the dual algorithm.
- DIPHA: We provide the script `dipha_reformat_output.m` to obtain the right format. The script takes as input the name of the binary file to which the ouput of the computations with DIPHA were stored.
- GUDHI: We provide the script `gudhi_reformat_output.m` to obtain the right format. The script takes as input the name of the text file to which the output of the computations with GUDHI are stored.
- JavaPlex: We wrote the three scripts `vietoris_rips_javaplex.m`, `lazy_witness_javaPlex.m`, and `witness_javaPlex.m`, which have been written to give this type of output.
- jHoles: We provide the script `jholes_reformat_output.m` to obtain the right format. The script takes as input the name of the text file to which the barcode intervals obtained with jHoles were stored.
- Perseus: The output is already in the right format.
- Ripser: The script `ripser_reformat_output.m` gives the right format.

Example:

```
>> dionysus_reformat_output('klein_bottle_25_output.txt','alpha')
```

creates the three files `klein_bottle_25_output_0.txt`, `klein_bottle_25_output_1.txt`, and further `klein_bottle_25_output_2.txt`, each storing intervals for PH in dimension 0, 1, and 2, respectively.

We can then plot barcodes using the script `plot_barcodes.m` and plot persistence diagrams using the script `plot_pdg.m`. Both scripts take as inputs (1) the name of a text file storing the intervals for PH in a certain dimension and (2) the title for the plot.

Example:

```
>>  plot_pdg('klein_bottle_25_output_1.txt','Klein bottle alpha dim 1')
```

produces the plot in Fig. 2(a) and saves it as a .pdf file `klein_bottle_25_output_1.pdf`.

Example:

```
>>  plot_barcodes('klein_bottle_25_output_1.txt','Klein bottle alpha dim 1')
```

produces the plot in Fig. 2(a) and saves it as a .pdf file `klein_bottle_25_output_1_barcodes.pdf`.

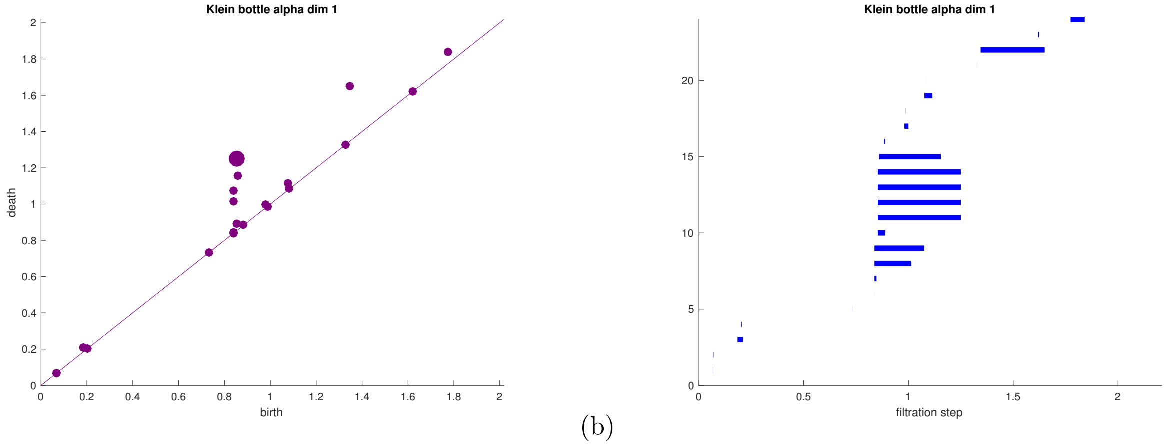

(a) (b)

Figure 2: Visualisation of output for the computation of PH in dimension 1 with the alpha complex (with DIONYSUS) for 25 points sampled uniformly at random form the Klein bottle: (a) persistence diagram and (b) barcode.

In the plots in Fig. 2, there are no infinite intervals, so we give an additional example to illustrate how infinite intervals are plotted with our scripts.

Example:

```
>> gudhi_reformat_output('klein_bottle_25_output.txt')
>> plot_barcodes('klein_bottle_25_output_0.txt','Klein bottle VR dim 0')
>> plot_pdg('klein_bottle_25_output_0.txt','Klein bottle VR dim 0')
```

produces the two plots in Fig. 3.

7 Statistical interpretation of barcodes

Once one has computed barcodes, one can interpret the results using available implementations of tools (such as bottleneck distance, Wasserstein distance, and persistence landscapes) that are useful for statistical assessment of barcodes. In this section, we give instructions for how to compute the bottleneck and Wasserstein distances with DIONYSUS and HERA. See the tutorial for the Persistence landscape toolbox [4] and the TDA package [5] for instructions on how to use these packages. For ease of reference, we recall the definition of Wasserstein distance from the main manuscript:

Definition 1 *Let $p \in [1, \infty]$. The pth Wasserstein distance between X and Y is defined as*

$$W_p[\mathrm{d}](X,Y) := \inf_{\phi\colon X \to Y} \left[\sum_{x \in X} \mathrm{d}[x, \phi(x)]^p \right]^{1/p}$$

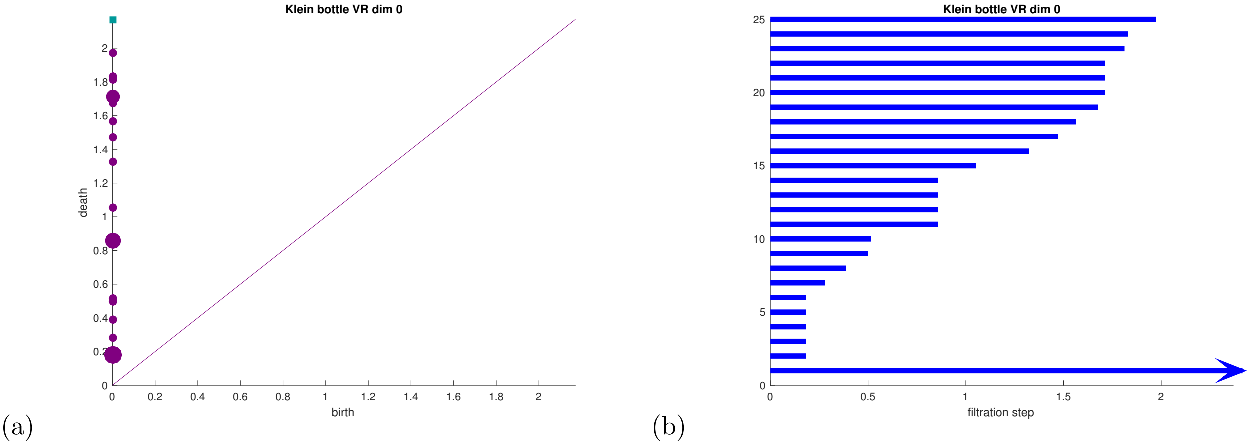

(a) (b)

Figure 3: Visualisation of output for the computation of PH in dimension 0 with the VR complex (with GUDHI) for 25 points sampled uniformly at random form the Klein bottle: (a) persistence diagram and (b) barcode. Infinite intervals are represented by a square in the persistence diagram, by an arrow in the barcode plot.

for $p \in [1, \infty)$ *and as*

$$W_\infty[\mathrm{d}](X,Y) := \inf_{\phi\colon X \to Y} \sup_{x \in X} \mathrm{d}[x, \phi(x)]$$

for $p = \infty$*, where* d *is a metric on* $\mathbb{R}^2$ *and* ϕ *ranges over all bijections from* X *to* Y*.*

7.1 Bottleneck distance

The bottleneck distance is the Wasserstein distance for $p = \infty$ and $\mathrm{d} = L_\infty$ (see Definition 1).

7.1.1 Dionysus

To compute the bottleneck distance between two barcodes, one can use the script `bottleneck-distance.cpp` (appropriately modified as explained in Section 2.1) in the Dionysus subdirectory `examples`. This script requires right endpoints of infinite intervals to be denoted by `inf` ; additionally, if there are intervals of length 0, the script will compute the wrong distance. To make sure that no intervals of length 0 are in the input files and that the intervals of infinite length are in the right format, one can use the script `bottleneck_dionysus.m`. This script takes as input two text files corresponding to two persistence diagrams in the unified format (see Section 6), with one interval per line, as follows:
`>> bottleneck_dionysus('pdg1','pdg2')`
and saves the persistence diagrams to two files called `diagram1.txt` and `diagram2.txt` to the current directory. Now one can compute the bottleneck distance as follows:

```
$ ./bottleneck-distance diagram1.txt diagram2.txt
```

7.1.2 Hera

To compute the bottleneck distance with Hera, one can use the script `bottleneck_dist` in the subdirectory `geom_bottleneck/build/example`. This script requires right endpoints of infinite intervals to be denoted by `-1` ; this corresponds to the convention in the unified format (see Section 6). One can compute the bottleneck distance as follows:

```
$ ./bottleneck_dist diagram1.txt diagram2.txt error
```

where `diagram1.txt` and `diagram2.txt` are two text files corresponding to two persistence diagrams in the

unified format, and `error` is a nonnegative real number that is an optional input argument. If `error` is nonzero, instead of the exact distance, an approximation to the bottleneck distance with relative error `error` will be computed. (See the explanation in [6].) This option can be useful when dealing with persistence diagrams that include many off-diagonal points, as it can speed up computations.

7.2 Wasserstein distance

With Dionysus, one can compute the Wasserstein distance for $\mathrm{d} = L_\infty$ and $p = 2$, and one can compute this distance for other values of p with a straightforward modification of the source code. With Hera, one can compute the Wasserstein distance for any choice of metric $\mathrm{d} = L_q$, with $q \in [1, \ldots, \infty]$, for any $p \in [1, \infty)$.

7.2.1 Dionysus

The script `bottleneck-distance.cpp` computes both the bottleneck distance and the Wasserstein distance for $\mathrm{d} = L_\infty$ and $p = 2$, so one can follow the instructions in 7.1.1 to compute the Wasserstein distance for these choices. If one wishes to compute the Wasserstein distance for other values of p, one has to modify the script `bottleneck-distance.cpp` as follows. Towards the end of the file, in the line

```
std::cout << "L2-Distance: " << wasserstein_distance(dgm1, dgm2, 2) << std::endl;
```

one can substitute the third input of the script `wasserstein_distance` with any number $p \in [1, \infty)$.

7.2.2 Hera

With Hera, one can compute the approximate Wasserstein distance discussed in [6]. One can use the script `wasserstein_dist` in the subdirectory `geom_matching/wasserstein/build`. This script requires right endpoints of infinite intervals to be denoted by `-1` ; this corresponds to the convention in the unified format (see Section 6). One can compute the approximate Wasserstein distance as follows:

```
$ ./wasserstein_dist power error distance diagram1.txt diagram2.txt
```

where `power` is the value for p, `error` is the relative error, `distance` is the value for q (where $\mathrm{d} = L_q$ is the employed distance), and `diagram1.txt` and `diagram2.txt` are two text files corresponding to two persistence diagrams in the unified format.

References

[1] Volvis repository. http://volvis.org.

[2] H. Adams and A. Tausz. JavaPlex tutorial. available at `https://github.com/appliedtopology/javaplex`.

[3] J. Binchi, E. Merelli, M. Rucco, G. Petri, and F. Vaccarino. jHoles: A tool for understanding biological complex networks via clique weight rank persistent homology. Electronic Notes in Theoretical Computer Science, 306(0):5–18, 2014. Proceedings of the 5th International Workshop on Interactions between Computer Science and Biology (CS2Bio14).

[4] P. Bubenik and P. Dłotko. A persistence landscapes toolbox for topological statistics. J. Symb. Comput., 78(C):91–114, January 2017.

[5] B. T. Fasy, J. Kim, F. Lecci, and C. Maria. Introduction to the R package TDA. ArXiv:1411.1830, November 2014.

[6] M. Kerber, D. Morozov, and A. Nigmetov. Geometry Helps to Compare Persistence Diagrams. ArXiv e-prints, June 2016. 1606.03357.

[7] H. Wagner, C. Chen, and E. Vuçini. Efficient computation of persistent homology for cubical data. In Ronald Peikert, Helwig Hauser, Hamish Carr, and Raphael Fuchs, editors, Topological Methods in Data Analysis and Visualization II, Mathematics and Visualization, pages 91–106. Springer Berlin Heidelberg, 2012.
